# Radiologic and histologic correlates of early interstitial lung changes in explant lungs

**DOI:** 10.1148/radiol.221145

**Published:** 2022-12-20

**Authors:** Stijn E. Verleden, Arno Vanstapel, Joseph Jacob, Tinne Goos, Jeroen Hendriks, Laurens J. Ceulemans, Dirk E. Van Raemdonck, Laurens De Sadeleer, Robin Vos, Johanna M Kwakkel-Van-Erp, Arne P. Neyrinck, Geert M. Verleden, Matthieu N. Boone, Wim Janssens, Els Wauters, Birgit Weynand, Danny D. Jonigk, Johny Verschakelen, Wim A. Wuyts

**Affiliations:** 1 Department of Chronic Diseases and Metabolism, BREATHE, KU Leuven, Belgium; 2 Department of ASTARC, University of Antwerp, Belgium; 3 Department of Respiratory Medicine, University hospital Antwerp, Belgium; 4 Department of Thoracic and Vascular Surgery, University hospital Antwerp, Belgium; 5 Department of Respiratory Medicine, University College London, UK; 6 Centre for Medical Image Computing, University College London, UK; 7 Department of Thoracic Surgery, University Hospital Leuven, Belgium; 8 Department of Cardiovascular Sciences, KU Leuven, Belgium; 9 Department of Physics and Astronomy, Ghent University, Ghent, Belgium; 10 Department of Imaging and Pathology, KU Leuven, Belgium; 11 Institute of Pathology, Hannover Medical School, Hanover, Germany. Biomedical Research in Endstage and Obstructive Lung Disease Hannover (BREATH), Member of the German Center for Lung Research (DZL), Hannover, Germany

## Abstract

**Background:**

Interstitial lung abnormalities (ILAs) reflect imaging features on lung CT scans that are compatible with (early) interstitial lung disease. Despite accumulating evidence regarding the incidence, risk factors and prognosis of ILAs, the histopathological correlates of ILAs remain elusive.

**Purpose:**

To determine the correlation between histopathological and radiological findings in CT-defined ILAs in human explant lungs.

**Materials and Methods:**

Explant lungs/lobes from participants with radiologically documented interstitial lung abnormalities were prospectively collected from 2010 to 2021. These specimens were air-inflated, frozen and scanned with CT and micro-CT (spatial resolution of 0.7 mm and 90 μm). Subsequently, the lungs were cut and sampled with core biopsies. At least 5 samples per lung underwent micro-CT and subsequent histopathological assessment with semi-quantitative remodeling scorings. Based on area-specific radiologic scoring, association between radiologic and histopathologic findings were assessed.

**Results:**

Eight lung explants from 6 donors (63% men) with a median age at explantation of 71 years (range, 60-83) were included (unused donor lungs, n=4; pre-emptive lobectomy for oncologic indications, n=2). Ex-vivo CT demonstrated ground-glass opacification, reticulation and bronchiectasis. Micro-CT and histopathology demonstrated that lung abnormalities were frequently paraseptal and associated with fibrosis and lymphocytic inflammation. Histopathology showed varying degrees of fibrosis in areas that appeared normal on CT. Regions of reticulation on CT scans generally had greater fibrosis on histopathological analysis. Vasculopathy and bronchiectasis were also often present on histopathological examination of lungs with ILAs. Fully developed fibroblastic foci were rarely observed.

**Conclusion:**

This study demonstrated direct histological correlates of CT-defined interstitial lung abnormalities.

## Abbreviations

ILA: Interstitial lung abnormality
ILD: Interstitial lung disease
IPF: Idiopathic pulmonary fibrosis

## Introduction

Interstitial lung abnormalities (ILAs) refer to specific CT findings potentially compatible with interstitial lung disease (ILD) in patients where morphological disease on CT imaging was not expected (1). A recent position paper by the Fleischner society put forward a more uniform description of ILAs as ground-glass or reticular abnormalities, lung distortion, traction bronchiectasis, honeycombing, and non-emphysematous cysts, encompassing at least 5% of any lung zone. The importance of ILAs has been demonstrated in several population-based and lung cancer screening studies. The incidence of ILAs is estimated to be 4-9% in smokers and 2-7% in non-smokers older than 60 years (2). Importantly, ILAs - mostly those with a subpleural distribution - are associated with subsequent progression to true ILD and higher mortality. According to the current literature, up to 20% of ILAs progress within 2 years, while over 40% progress within 5 years (2).

While knowledge on the incidence, risk factors and prognosis of ILAs has markedly improved recently (1,2), the exact morphological and histopathological correlates of these imaging features remain elusive. Miller et al. found ILAs in 26 (6%) of 424 tumor resection specimens, of which 3 (12%) demonstrated a centrilobular distribution, 17 (65%) were subpleural, and 6 (23%) were of mixed distribution (3). Hung et al. (4) analyzed 397 lung tumor resection specimens where 101 (25%) patients showed parenchymal abnormalities. Of these, 41 (10%) were classified as fibrotic remodeling and mostly corresponded to smoking-related interstitial fibrosis. The presence of histopathological abnormalities also correlated with the presence of ILAs on CT imaging. To our knowledge, no studies that have specifically investigated the correlation between ILA patterns on CT and histology.

A protocol to perform accurate matching of in-vivo CT with histology using ex-vivo CT and micro-CT imaging as intermediate steps has been developed (5–8). In idiopathic pulmonary fibrosis (IPF), the inherent spatial heterogeneity associated with usual interstitial pneumonia, the typical radiological pattern observed in IPF, was exploited to compare areas of minimal and fully developed fibrosis. Interestingly, fibrosis and cyst formation manifested at the periphery of the pulmonary lobule and progressively extended towards the core of this anatomical compartment (9). A major limitation of these previous studies was that all lungs were end-stage, and it remains unknown if these changes are observed at the early disease stages. Therefore, the purpose of this study was to investigate the correlations between histopathological and radiological findings in whole lungs/lobes with CT-defined ILAs.

## Material and Methods

### Study Participants and Data

In this prospective institutional review board (IRB)-approved study, all participants provided written informed consent. Unused donor lungs deemed to be of insufficient quality were collected from 2010-2021 (IRB S52174, S61653). For most of these specimens, in vivo CT scans were unavailable. Lobar specimens following tumor resection in cases where the suspicious nodule was initially wedge resected and found to be malignant were also collected in 2020 (IRB approval S63093). Unused donor lungs and lobar lung resection specimens were also collected from a second site in 2021 (IRB B3002021000073).

### CT and Micro-CT Acquisition

Collected lungs/lobes were air-inflated at 30 cm water pressure and frozen in liquid nitrogen following gentle deflation to 10 cm water pressure. An ex-vivo CT-scan (Siemens Somatom or GE Healthcare, 120 kvp, 10 mas, FOV 300 mm, collimation 0.6 mm, 1 mm slice thickness with an increment of 0.7 mm) was then performed. Following the ex-vivo CT scan, a whole lung micro-CT scan (80 kvp, 0.300 mas, FOV 257 mm, voxel size 0.09 mm, Hector (10), or Unitom XL) was taken in frozen state to improve the accuracy of matching between ex-vivo CT and specimen micro-CT. Subsequently, the lungs were sliced with a bandsaw at 2 cm slice thickness and cores with a diameter of 14 to 20 mm were systematically extracted from these samples. A photograph before and after the samples were taken ensures accurate matching between in-vivo CT (if available), ex-vivo CT, whole lung micro-CT and core micro-CT and histology. Micro-CT was performed at -30°C (Bruker Skyscan 1272, 10μm voxel size, 0.5 rotation step). Cores were selected to represent the spatial heterogeneity in the lung in areas surrounding the CT identified ILA’s, ranging from no visible abnormalities to obvious opacities visible on ex-vivo CT. Five areas per lung were analyzed, except for one case where 6 areas were investigated because there was more spatial heterogeneity of disease. These samples subsequently underwent fixation with 6% paraformaldehyde, were dehydrated and paraffin embedded. The study design and workflow are further illustrated in figure 2.

### CT and Micro-CT Assessment

The ex-vivo CT scan was assessed by two experienced thoracic radiologists blinded for all participants information (JJ 15 years of CT expertise and JV 34 years of CT experience), and a systematic description of the general radiologic pattern (reticulation, ground-glass opacification, honeycombing, bronchiectasis) and overall extent of parenchymal remodeling was provided. The spatial heterogeneity of disease in areas on ex-vivo CT from which the cores were later taken were assessed by two independent experienced thoracic radiologist (JJ 15 years of expertise and JV 34 years of experience): one main observer and one to assess consistency of the scoring. The degree of normal tissue, reticulation and ground-glass opacification were assessed per individual lung core using a semi-quantitative scale (0-100% estimated to the nearest 5%), while the degree of bronchiectasis was assessed as present/absent. MicroCT based segmentation of selected airways was performed using ITK-SNAP.

### Histopathological Assessment

Histologic slices were cut and stained with hematoxylin & eosin, trichome and Von Gieson’s stain. All slices were investigated by a pulmonary pathologist experienced in non-neoplastic lung pathology (DDJ, 16 years of experience) blinded for clinical information and were assessed for the following parameters: presence, location (para-septal, peri-bronchial and interstitial) and severity/amount of fibrosis (0- no evidence of fibrosis; 1- Mild fibrosis: no clear organization of fibrosis is observed; 2- Moderate fibrosis: beginning organization of fibrosis; 3- Severe fibrosis: fibrosis as seen in end-stage lung disease); presence of vasculopathy (i.e. abnormalities in the vasculature such as intimal thickening, yes/no); presence of fibroblast foci (yes/no, if yes fully developed or incomplete); location (para-septal, peri-bronchial and interstitial) and type of predominant inflammation (neutrophilic, lymphocytic, macrophages); presence of bronchiectasis; and other potentially relevant findings.

### Statistical Analysis

Results are expressed as either mean or median. Variables were dichotomized based on predominant local CT pattern (≥70% healthy; ≥50% reticulation; ≥ 40% ground glass opacification vs lower) and compared using a Mann Whitney U test. *P* <.05 was considered to indicate a statistically significant difference. All analyses were performed using GraphPad Prism 9.0 (GraphPad Software).

## Results

### Study Participants and Data

**Figure 1:**
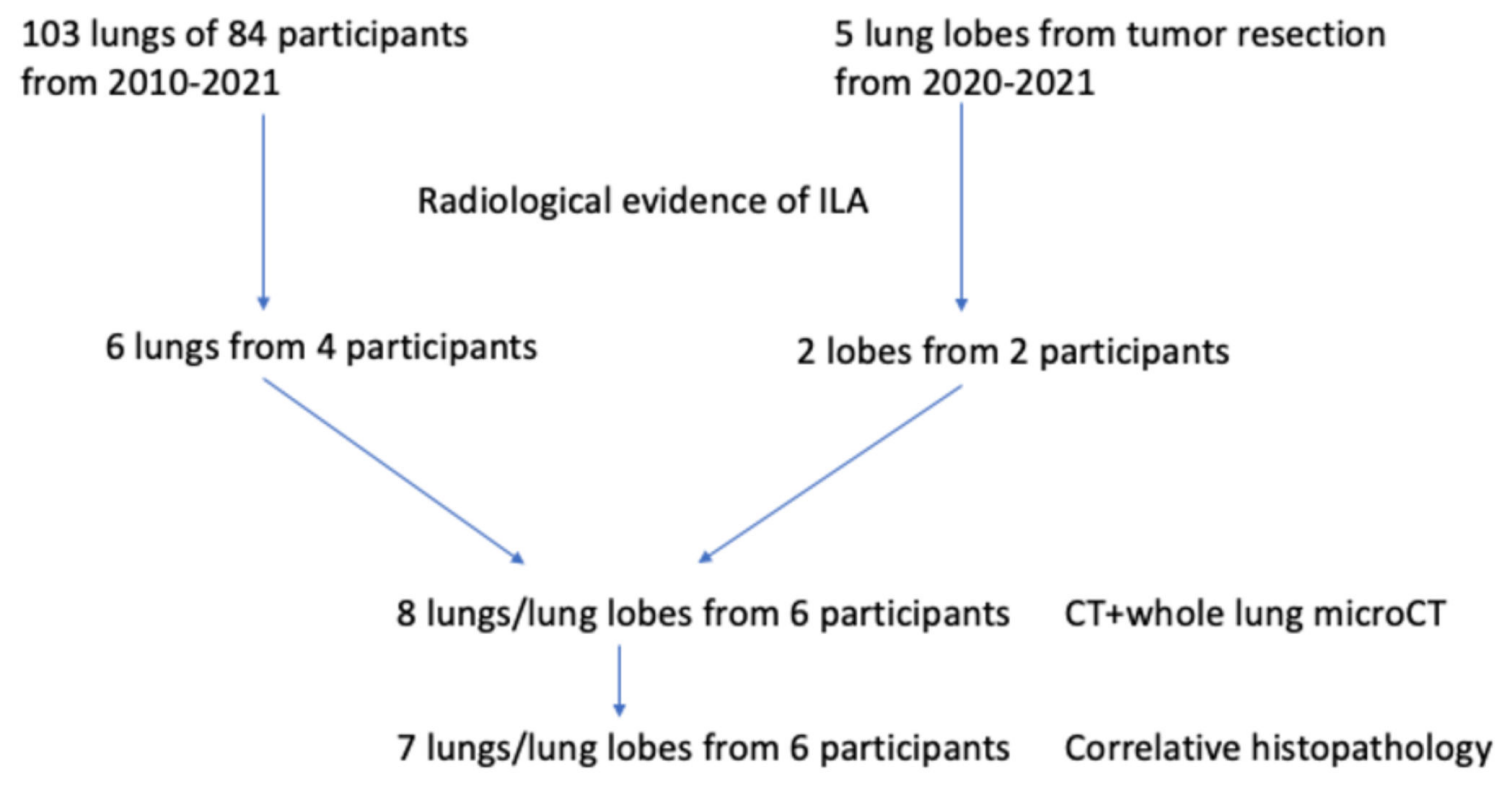
Flow chart of participants inclusion. ILA = interstitial lung abnormality

**Figure 2:**
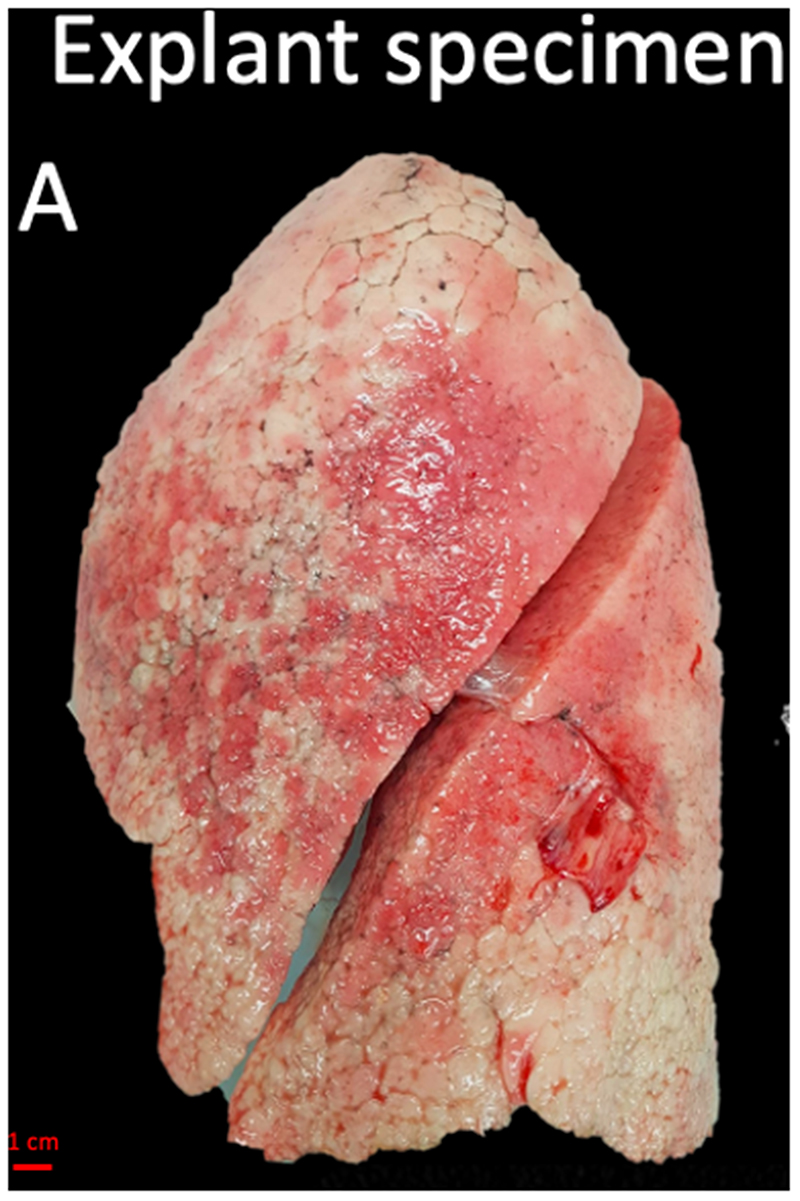

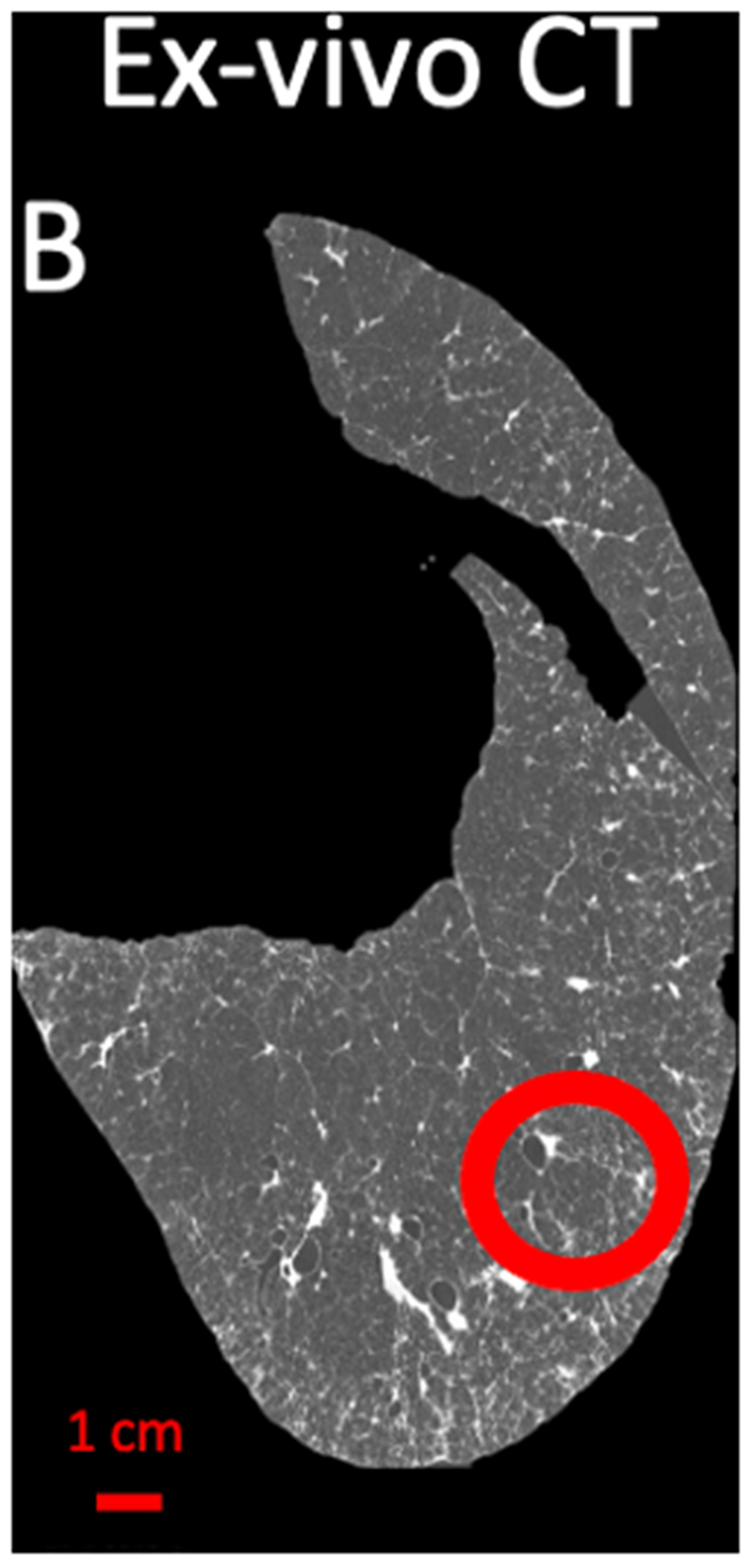

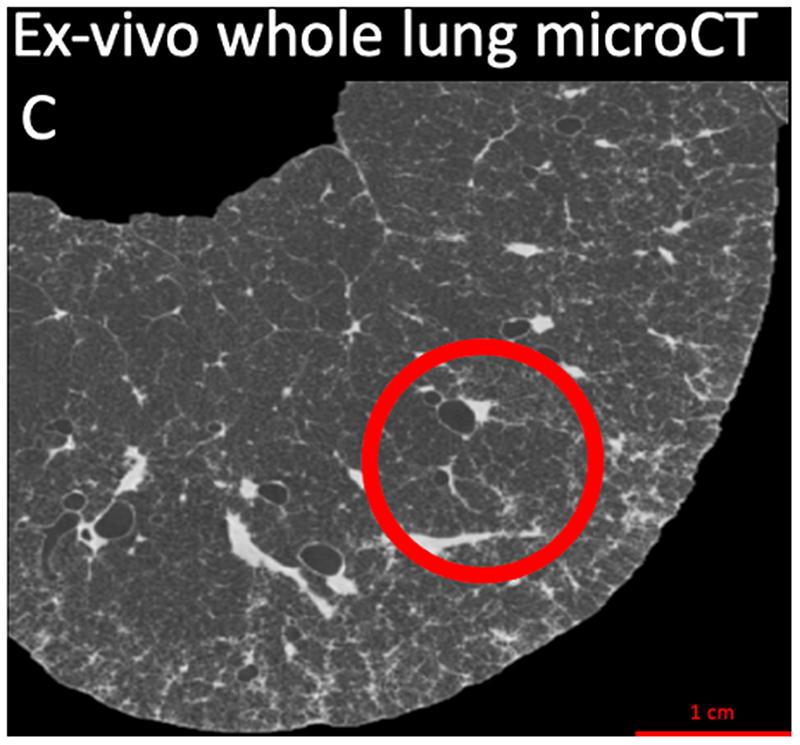

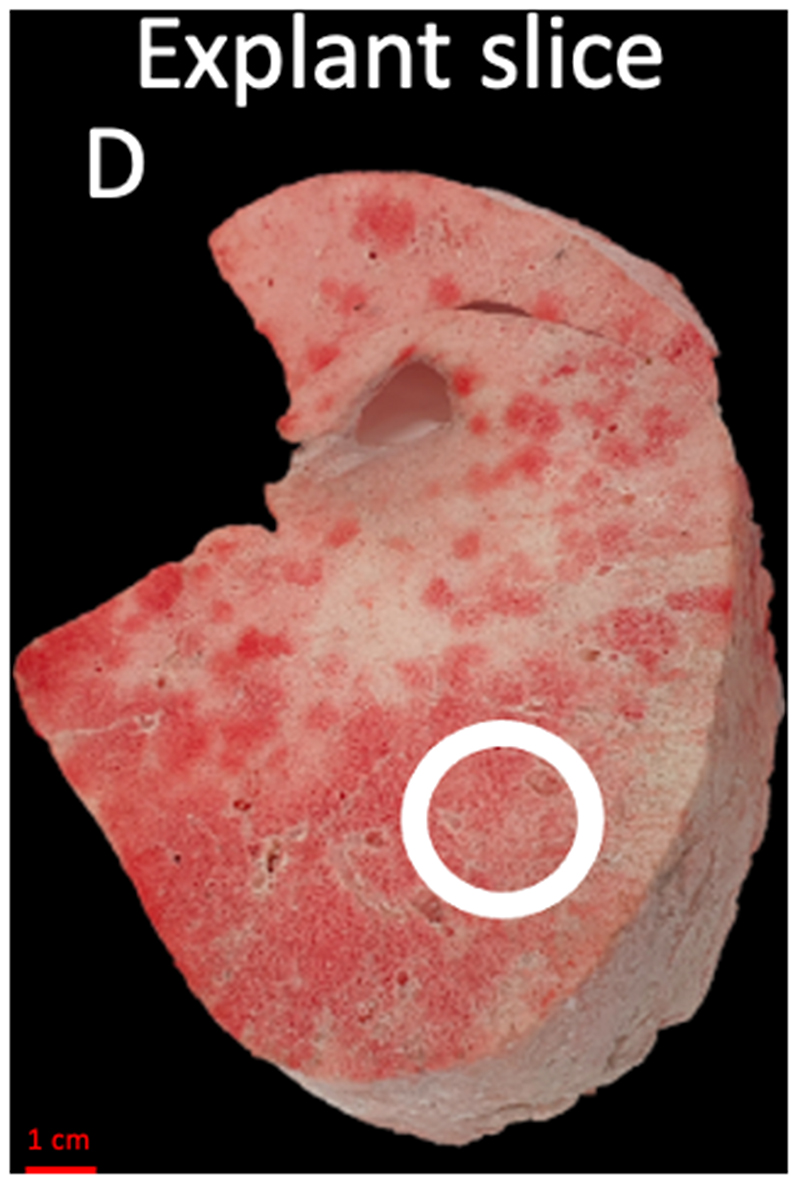

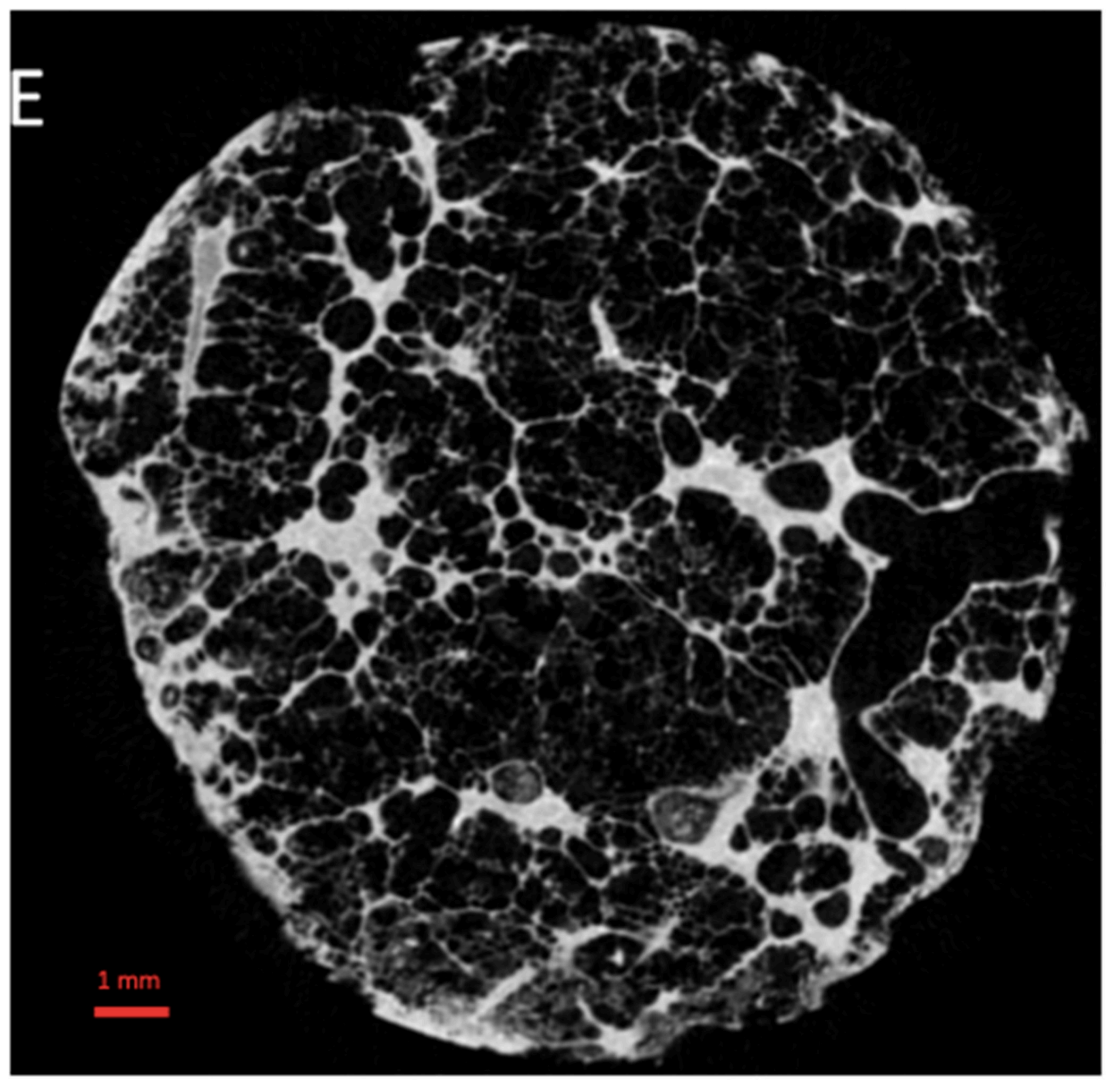

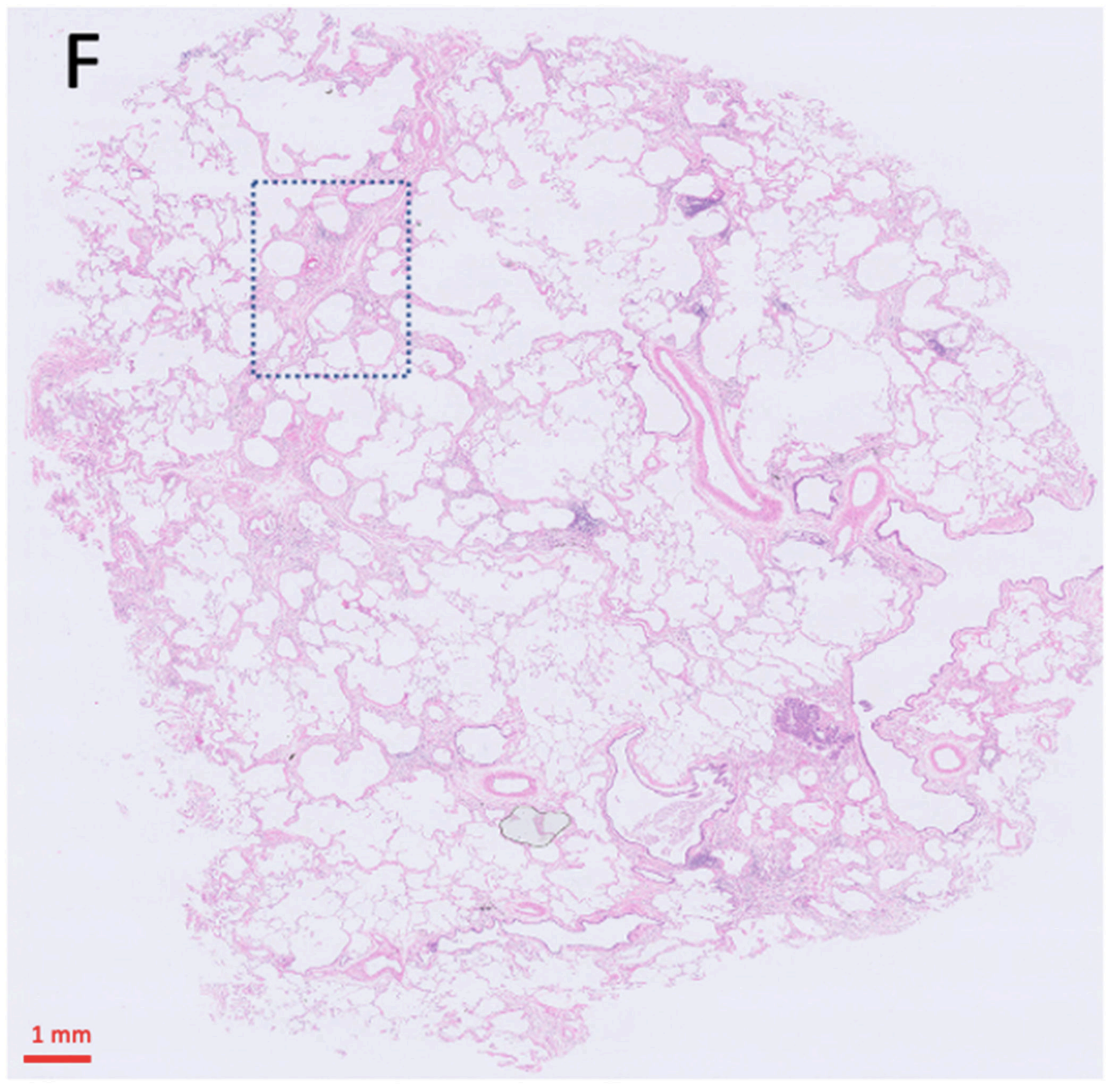

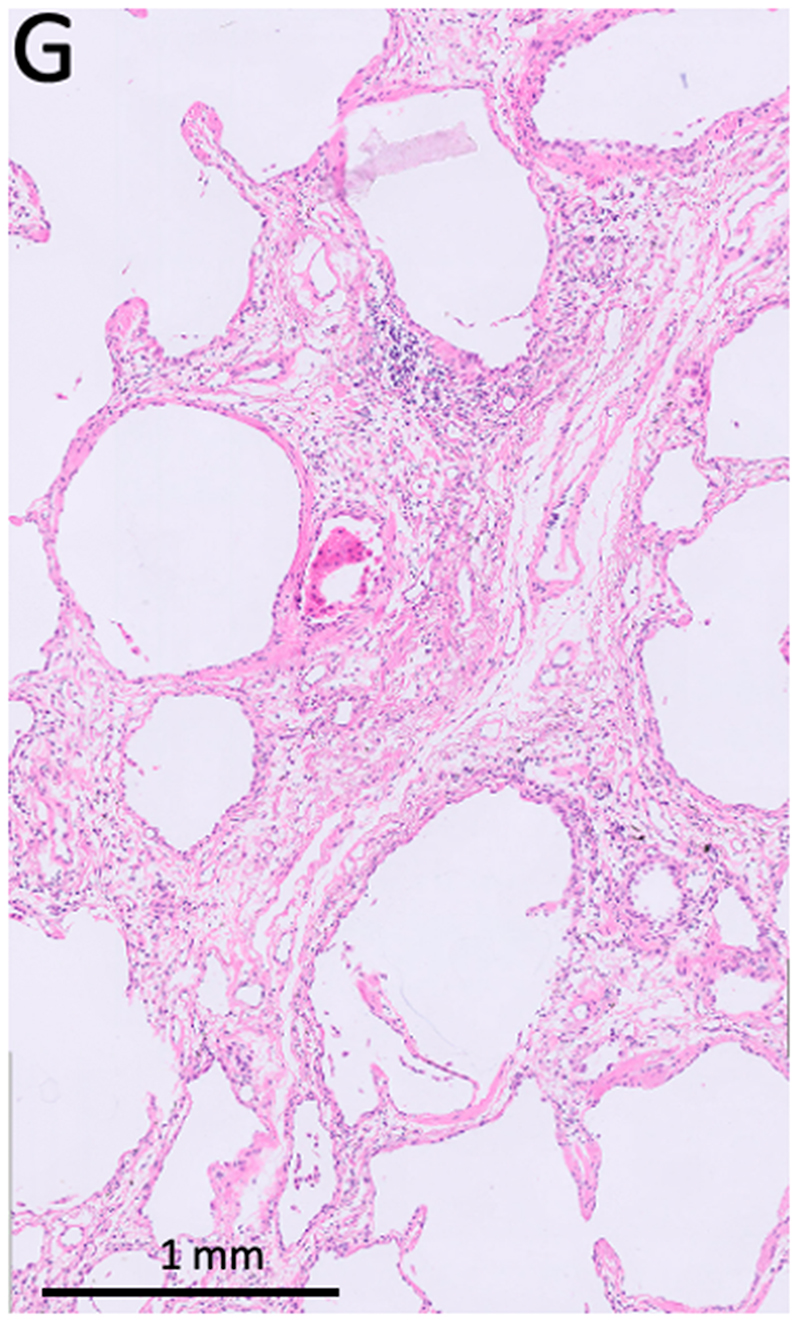
Study design. (A) The explant lung was frozen solid in liquid nitrogen fumes. (B) An ex-vivo noncontrast CT scan was obtained of the specimen while it was frozen (axial view). (C) For better spatial resolution, a whole lung micro-CT was obtained. (D) The lung was sliced transversally in 2 cm slices. (E) Micro-CT scan of a core sample indicated with the circle in B, C, and D was performed. (F) Matched H&E image (5x magnification) at the same location of the micro-CT scan. (G) High magnification H&E image (20x magnification) view showing para-septal fibrosis

A total of 103 unused donor lungs/lobes from 84 participants were collected since 2010; their ex vivo CT scan was assessed, and 6 lungs of 4 participants showed evidence of ILAs on ex-vivo CT while medical history did not reveal any known pulmonary disease prior to organ donation. Five lobes from tumor resection surgery were collected since 2020, of which two showed signs of ILAs in participants who were not known to have chronic lung disease and where an incidental nodule was found. Due to technical limitations, in one lung of the included lung pairs derived from an unused donor, ex-vivo CT analysis alone was undertaken with no histopathological analysis performed. The flowchart is shown in Figure 1. Our study included eight explant specimens from a total of 6 participants (unused donor lungs, n=6 from 4 participants; tumor resection specimens, n=2 from 2 participants). The median age at explantation was 71 years (range, 60-83). Characteristics are summarized in Table 1.

### Radiologic Assessment

A varying degree of sub-pleural ground glass opacification and reticulation was found in all lung as confirmed by both observers (Supplemental figure 1). Patterns compatible with lung damage were more pronounced in the lower lobes in five of the eight specimens (three participants, supplemental figure 1A, C, D, G, H), while the opacities did not show a basal predominance in the other three specimens (two participants, supplemental figure 1B, E, F). Two specimens were only mildly affected (5-10% of lung volume, supplemental figure 1A, H), four were moderately affected (10-20% of lung volume, supplemental figure 1B, C, D, G), and two specimens were severely affected (20-30% of lung volume, supplemental figure 1E, F). Besides ground-glass opacification and reticulation, five specimens showed evidence of bronchiectasis (all specimens according to the second radiologist), and two had some degree of emphysema as characterized by the presence of sub-pleural bullae or para-septal emphysema (Table 1).

### Histopathologic Assessment

**Figure 3:**
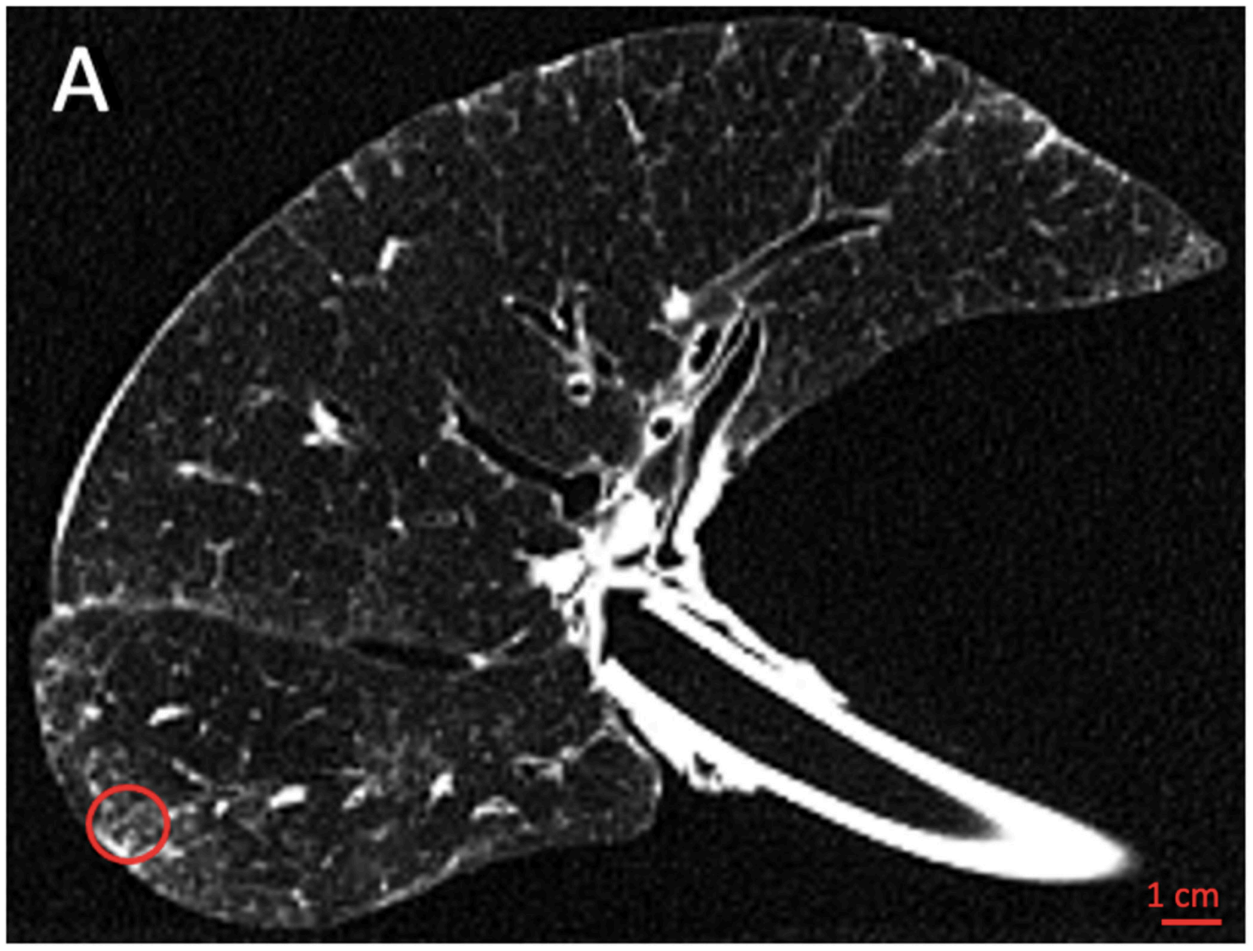

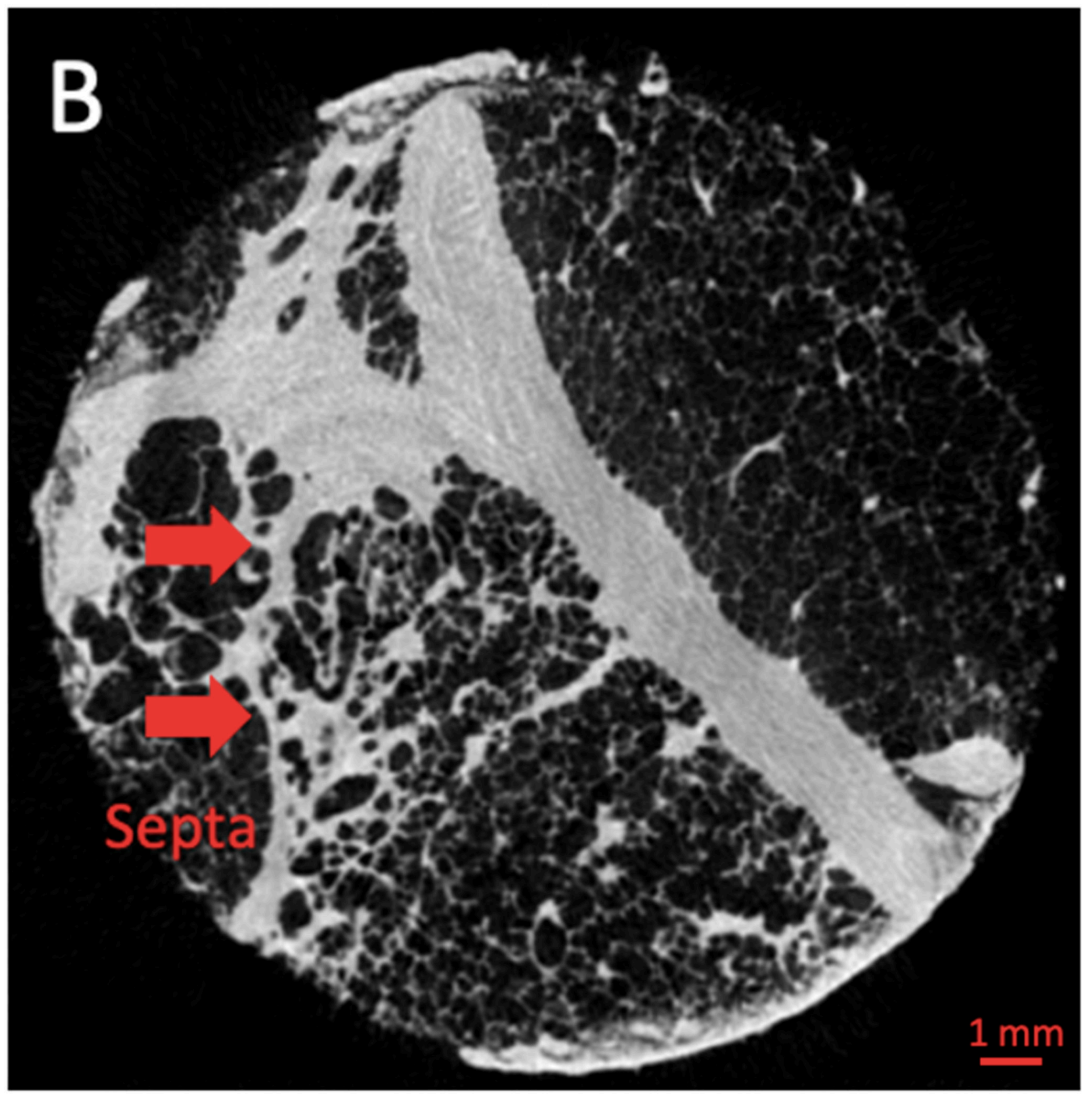

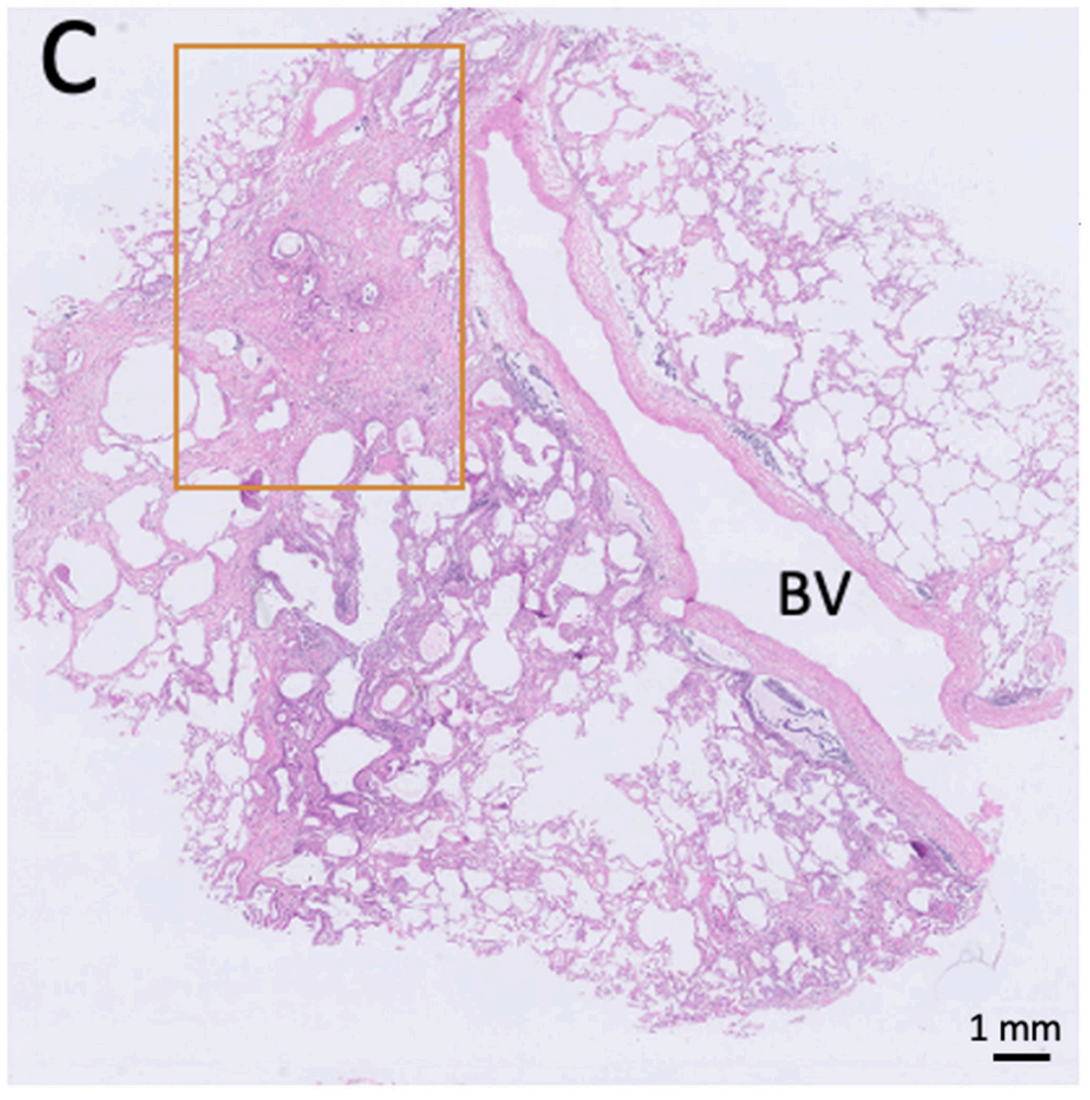

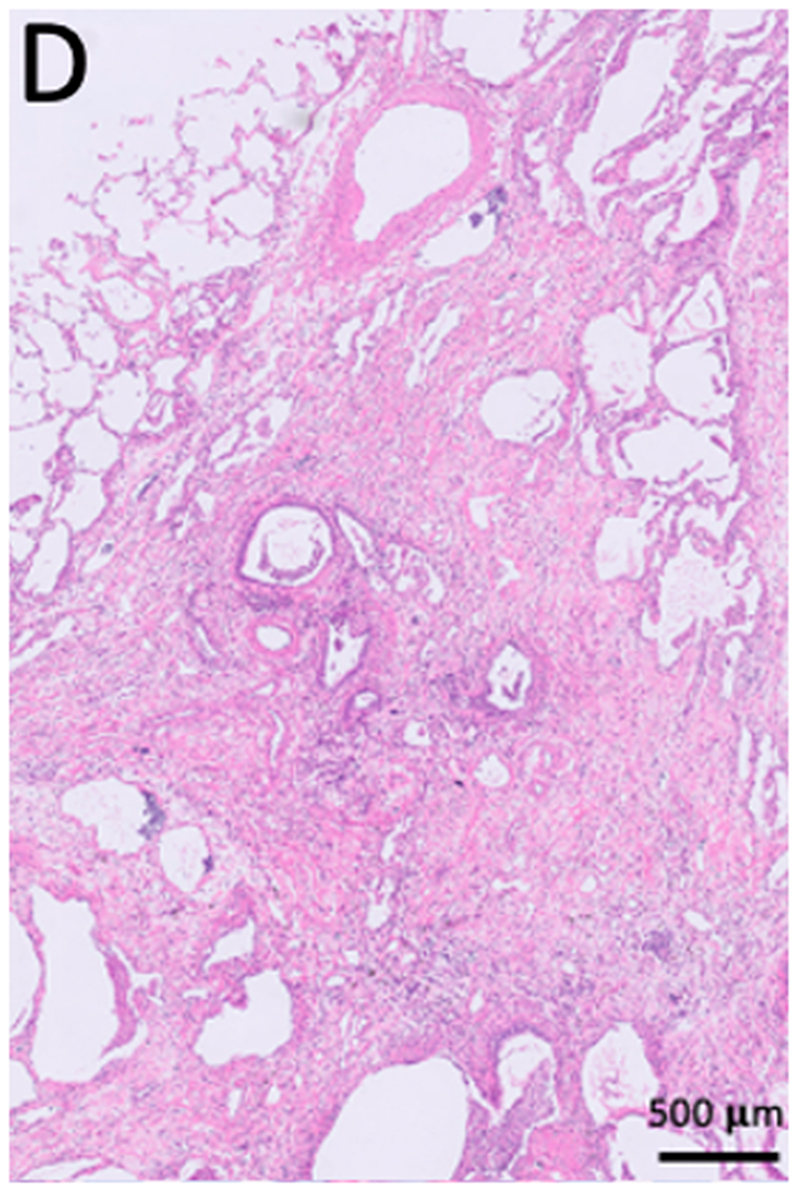

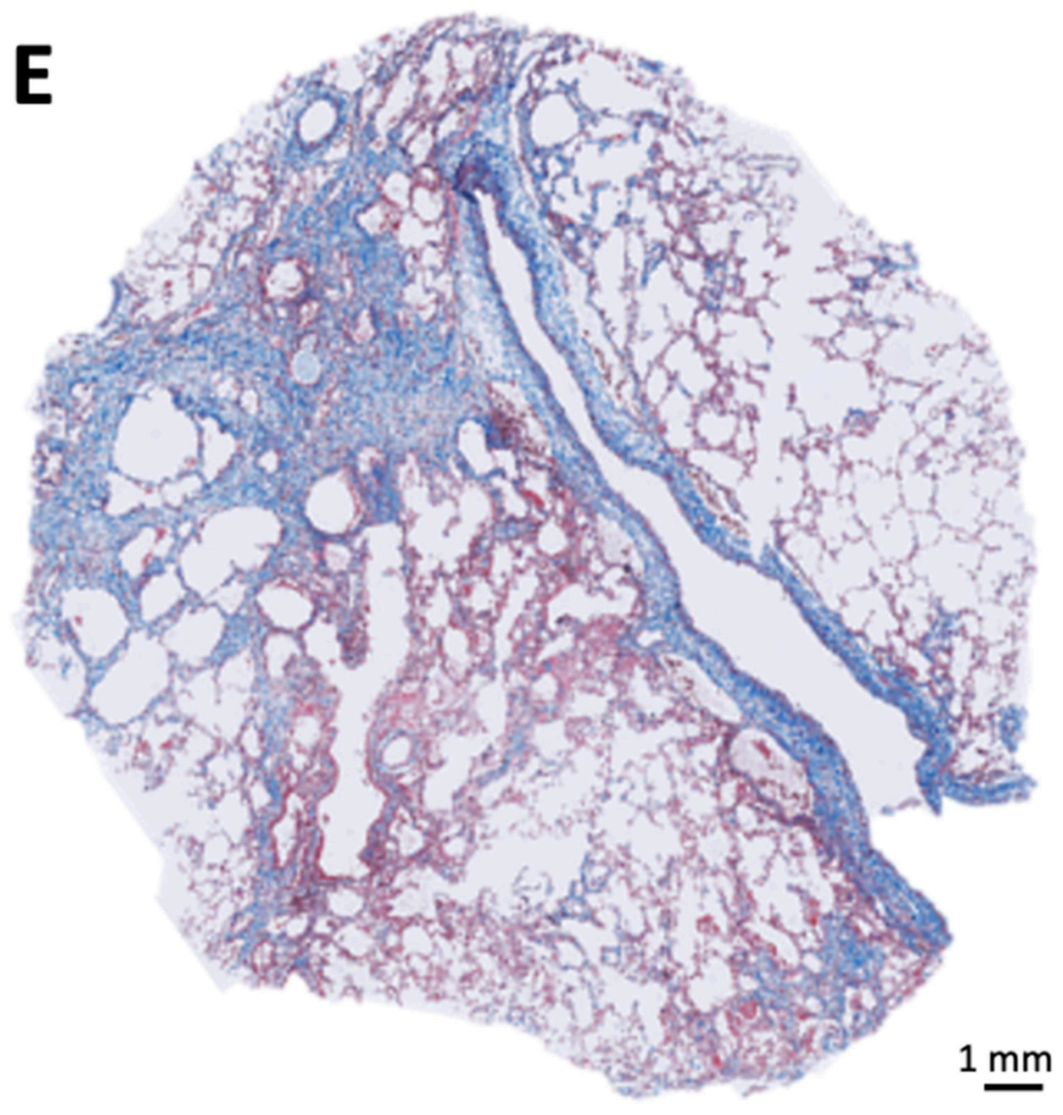

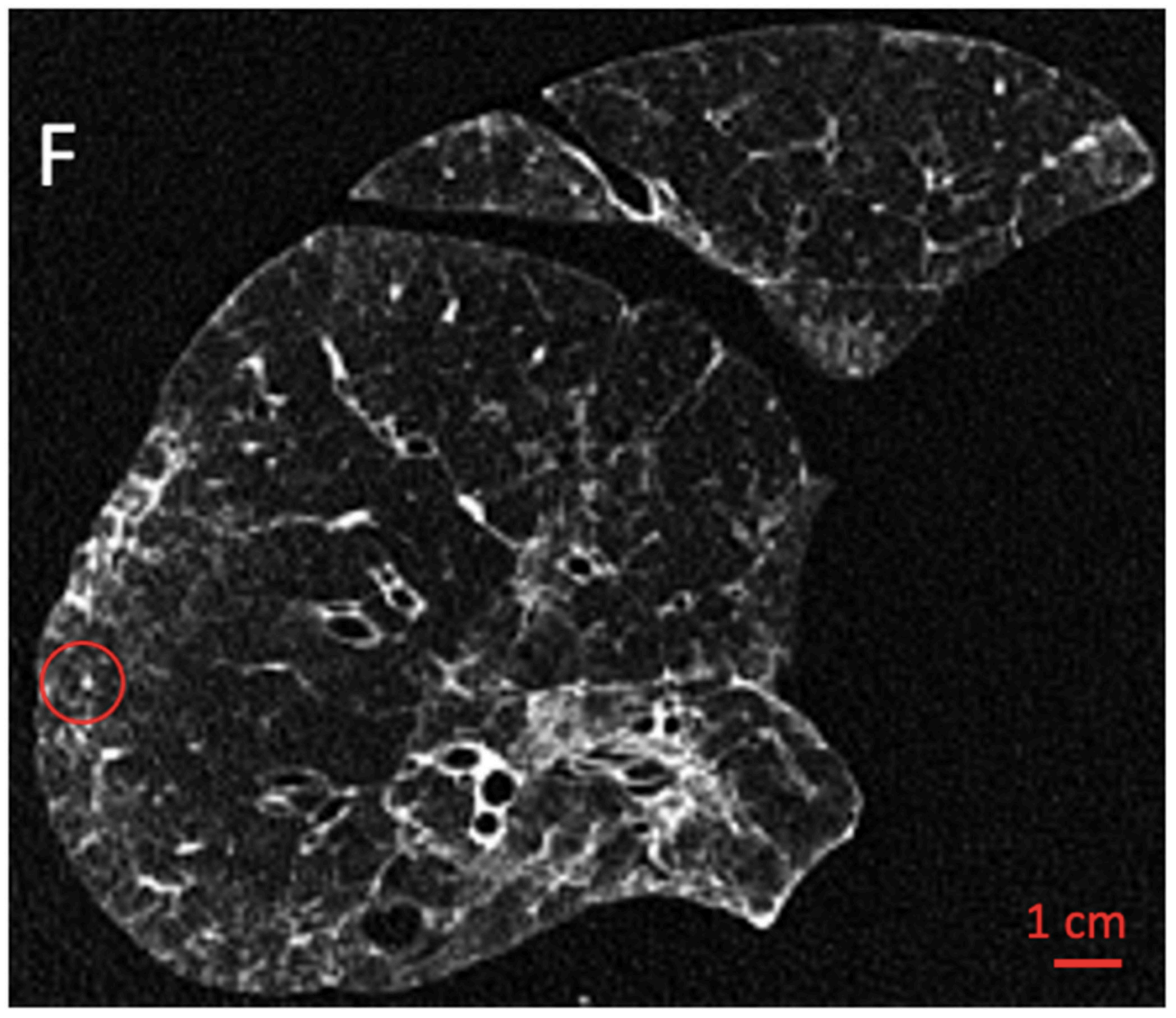

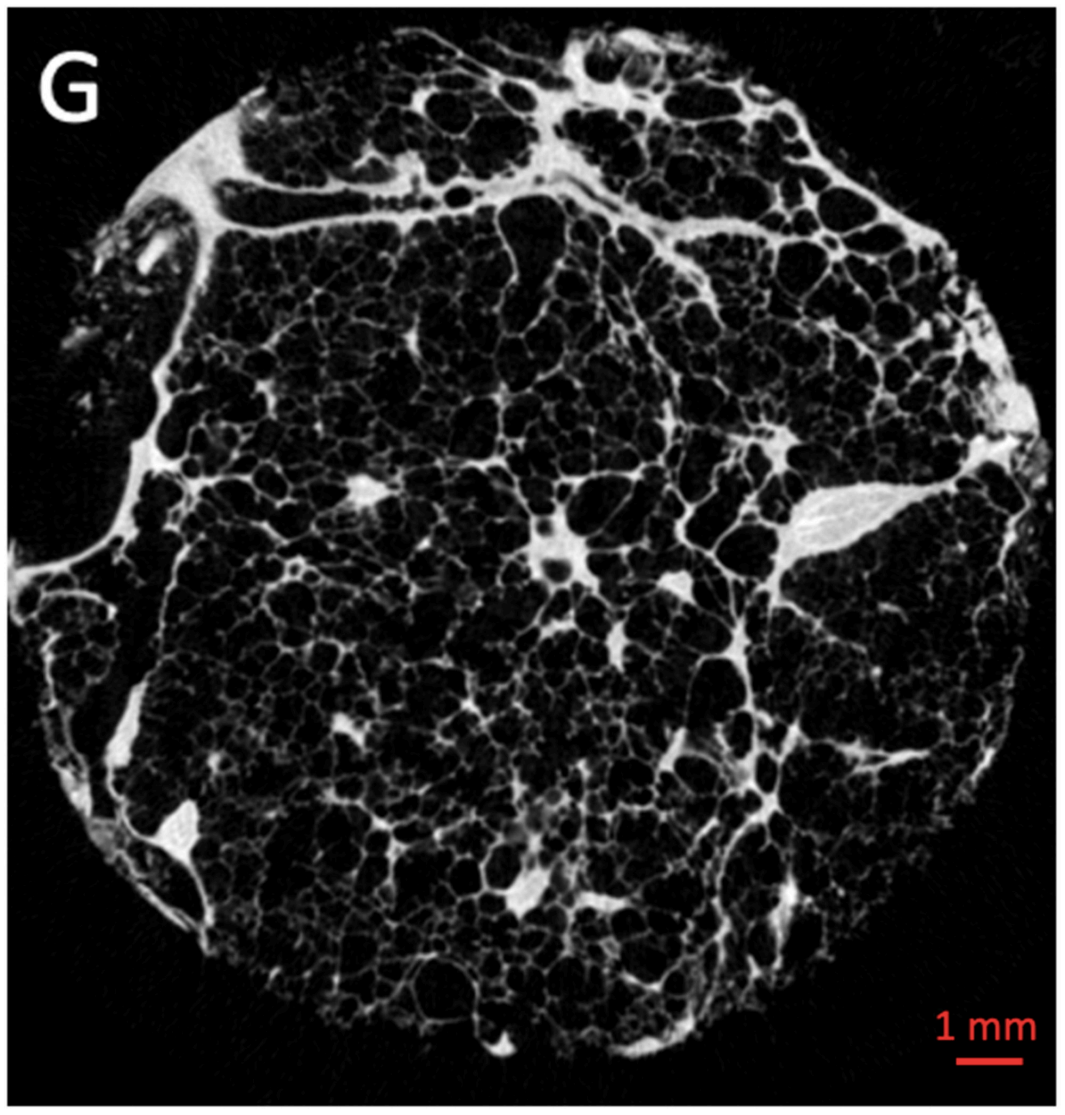

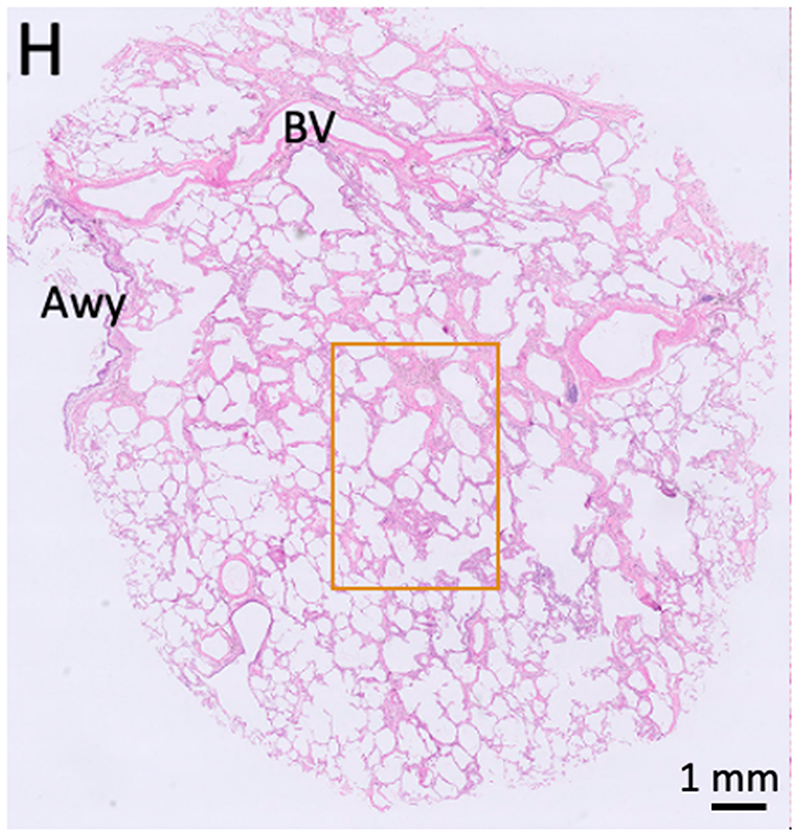

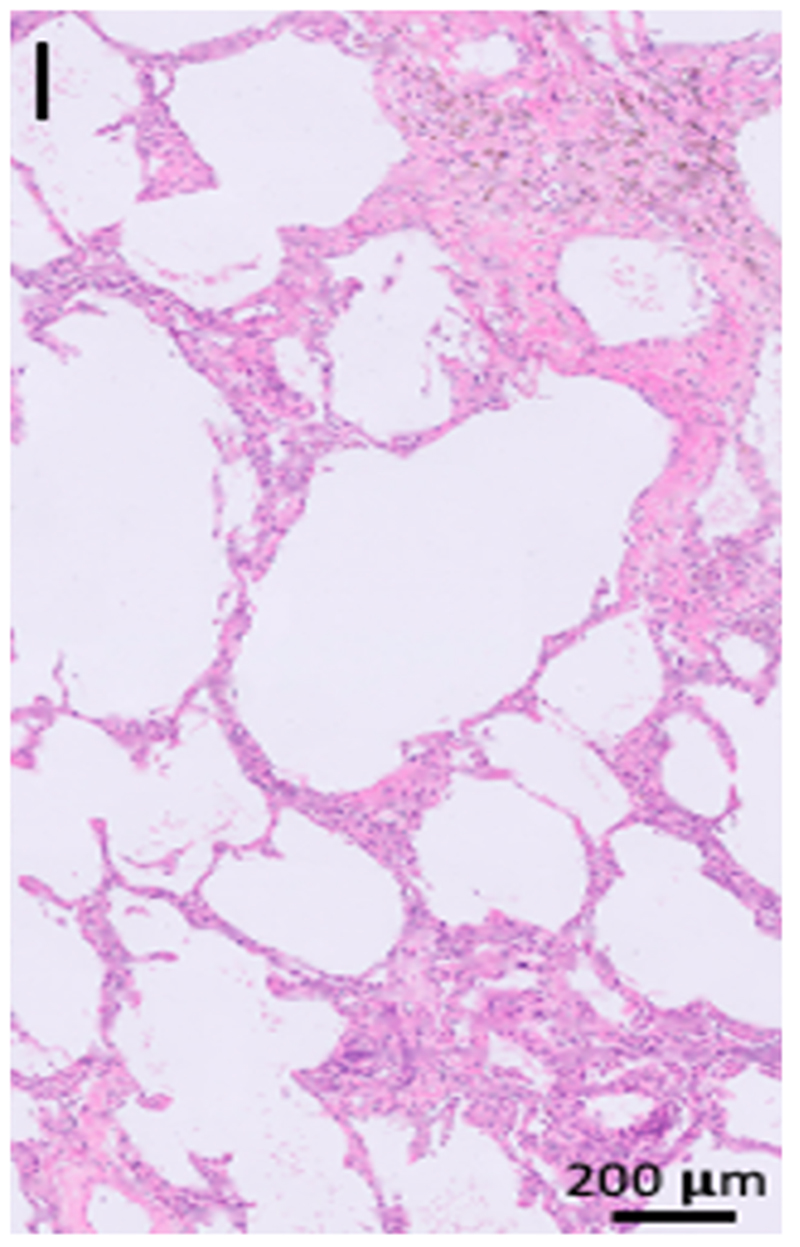

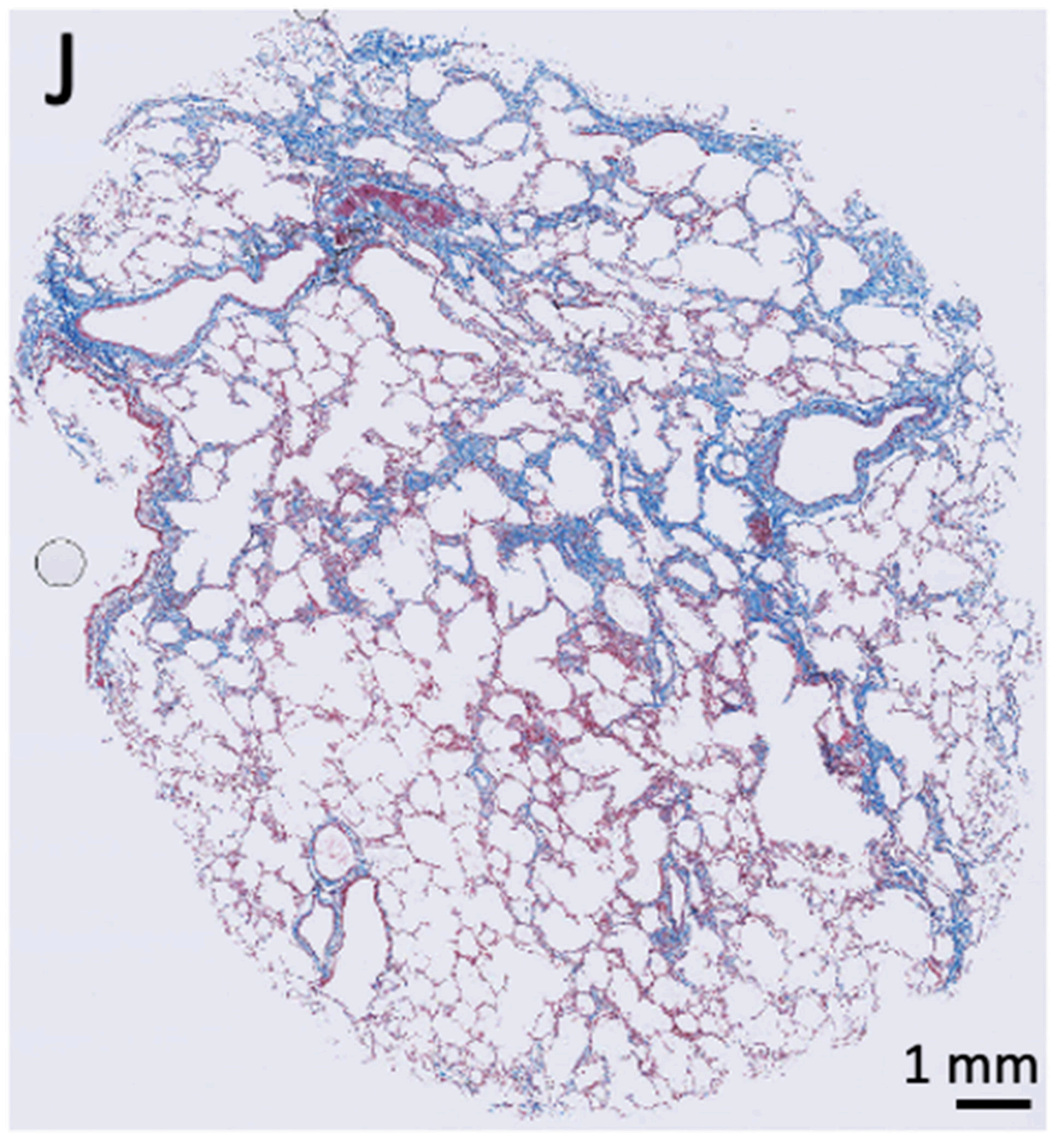
Illustrative example of sampled lung areas with ex-vivo CT, micro-CT and histology. (A) Illustrative example of an ex vivo lung CT scan (axial view, no contrast) which was considered highly abnormal (25% healthy, 55% reticulation, 20% ground-glass opacification) (B-E). Micro-CT, histopathology (hematoxylin and eosin (H&E) and trichrome staining) of the area highlighted with a circle in A showing para-septal and interstitial fibrotic changes (5× magnification for C-E, 20× for D). (F) Illustrative example of an ex-vivo lung CT scan (no contrast) which was considered grossly healthy (80%) with mild ground-glass opacification (15%) and limited reticulation (5%) (G-J). Micro-CT and histopathology (H&E and trichrome staining) of the matched location (circle in F) showing para-septal and interstitial fibrosis. (5× magnification for H-J, 20× for I). Awy = airway, BV = blood vessel

A total of 36 samples from seven specimens were separately assessed based on the ex-vivo CT scan and showed median (range) values of 40% (25-75%), 25% (15-40%) and 20% (10-30%) for normal tissue, reticulation, and ground-glass opacification, respectively. The main findings are summarized in Table 2, with representative examples shown in Figure 3.

Within the 36 samples, 30 (83%) showed some degree of fibrosis on histopathological assessment (mild, n=20; moderate, n=10). This was exclusively organized peri-bronchially in two of 36 samples (6%) and exclusively para-septally in six samples (17%), but never exclusively interstitial. A combination of para-septal and interstitial changes was observed in nine (25%) of the 36 total samples, and a combination of peri-bronchial and para-septal changes was observed in three samples (8%). In ten samples (28%), fibrosis was present in all compartments (peri-bronchial, para-septal and interstitial). Taken together, para-septal fibrosis was the most frequent distribution observed in 28/36 (78%) samples, followed by interstitial fibrosis in 19/36 (53%) samples and peri-bronchial fibrosis in 15/36 samples (42%). Fully developed fibroblast foci were present in only three samples (8%) of 3 different lungs (3 participants), with incomplete fibroblast foci in an additional 4 samples (11%). These foci were exclusively found in association with fibrosis (mild, n=5; moderate n=2) and in areas with lymphocytic inflammation. Interestingly vasculopathy was present in six of the 7 samples with fibroblast foci.

Inflammation was present in 31/36 samples (86%) and was almost exclusively dominated by lymphocytes (peri-bronchial, n=2; para-septal, n= 5; interstitial, n=7; peri-bronchial and para-septal, n=2; para-septal and interstitial, n= 10; and peri-bronchial, para-septal and interstitial, n=5).

Lymphocytic inflammation was present in 4/6 (67%) samples without fibrosis, in 17/20 (85%) samples with mild fibrosis and in all 10 samples with moderate fibrosis (100%). Neutrophilic inflammation was not observed while one sample showed a desquamative interstitial pneumonia characterized by macrophage inflammation.

Vasculopathy was present in 4 lungs of 3 participants; 3/6 (50%) samples without concomitant fibrosis, 5/20 samples (25%) with mild fibrosis, and 4/10 samples (40%) with moderate fibrosis.

**Figure 4:**
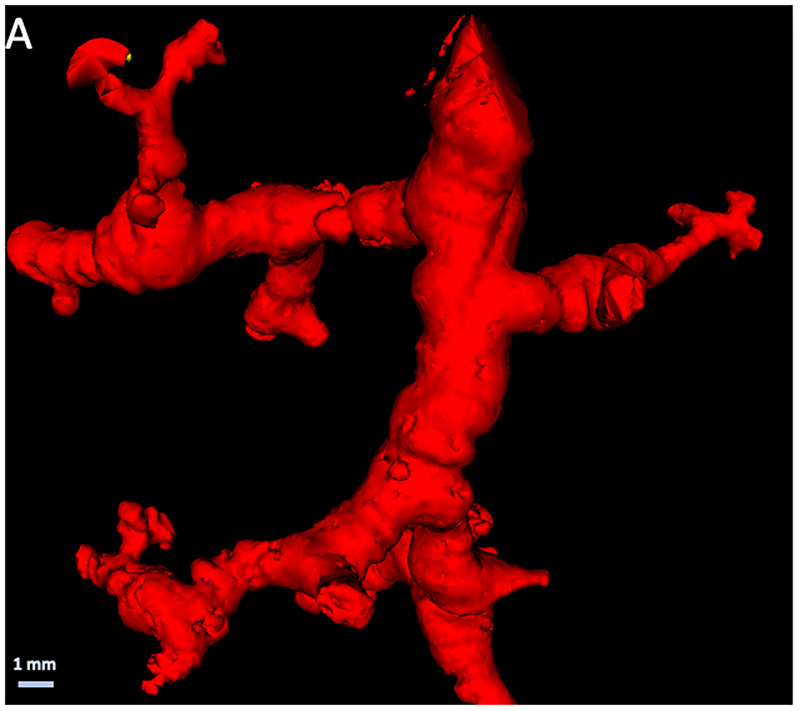

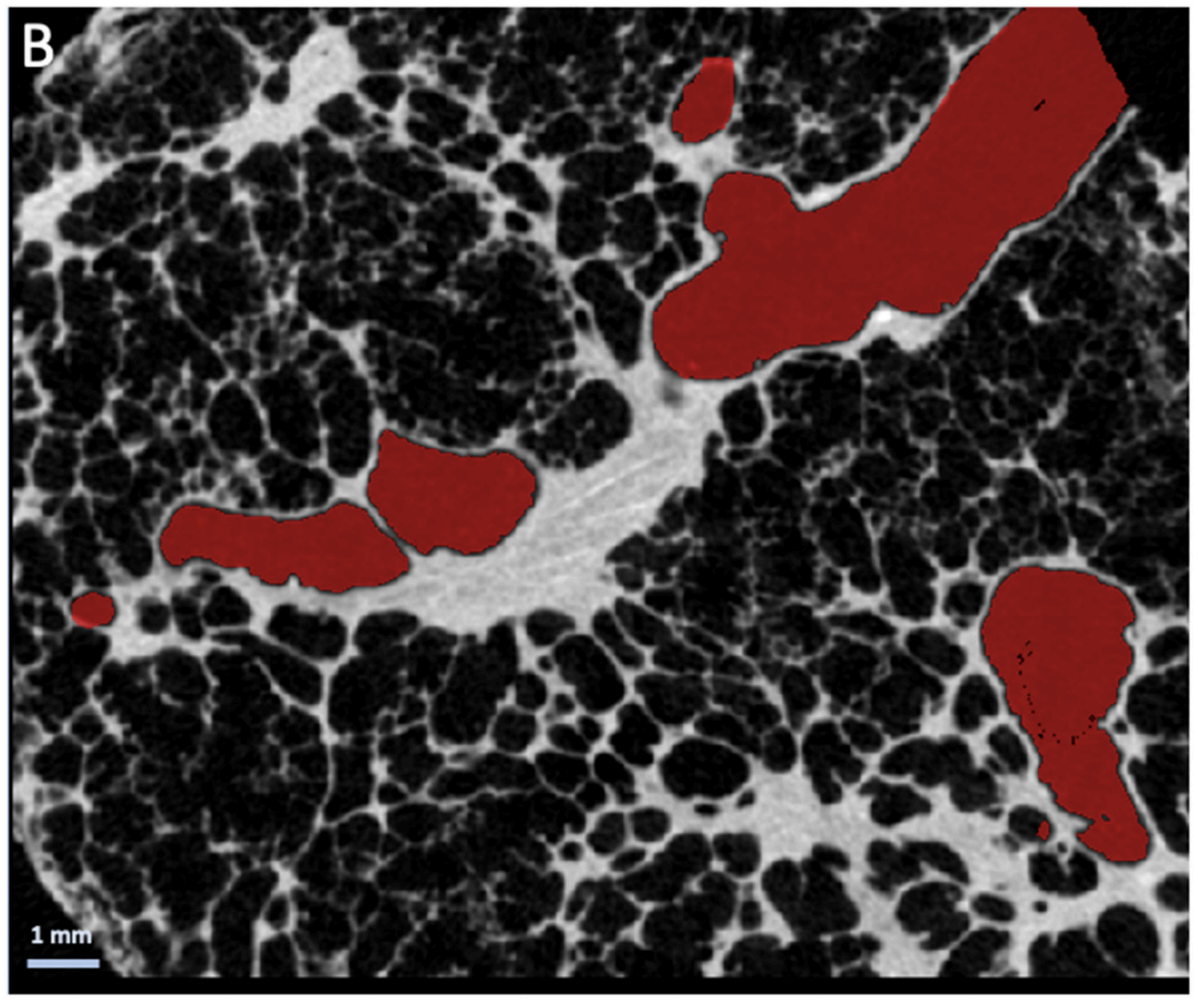
(A) Three-dimensional reconstruction of distal airway tree based on specimen micro-CT scans demonstrating typical tortuous and distorted airways. (B) Cross-sectional micro-CT panel demonstrating the absence of overt fibrosis in the vicinity of the airway (airway is highlighted in red).

Histopathologic evidence of bronchiectasis was observed in 14/36 samples (39%) and was present in 6 of the 7 studied lungs (5 participants). Bronchiectasis was found in areas without histopathologic evidence of fibrosis (n=1, 17% of samples without fibrosis), areas with mild fibrosis (n=9, 45% of samples with mild fibrosis) and in areas with moderate fibrosis (n=4, 40% of samples with moderate fibrosis). An example of airway distortion in the peripheral pre-terminal airways, observed in the absence of severe interstitial fibrosis, in lungs with ILAs is shown in Figure 4.

### Correlation Between Radiology and Histopathology

Core-specific information concerning radiology and histology is provided in the online supplement. There was respectively 80%, 86% and 80% consistency in the assessment of healthy tissue, reticulation and ground glass opacification between the 2 radiologist observers. Regions that showed limited evidence of remodeling based on radiological scoring of the ex-vivo CT (≥70% core volume of healthy parenchyma, n=11/36) nevertheless contained considerable fibrosis on histopathology (mild fibrosis, n=8), indicating that CT underestimated the degree of fibrosis in relatively normal appearing lung (organization para-septal, n=7; interstitial, n=5; peri-bronchial, n=4). A representative example is shown in supplemental figure 2. This was also accompanied by lymphocytic inflammation in nine of eleven (82%) samples that were relatively normal. In samples predominantly affected by reticulation (≥50% core volume, n=7/36), more severe fibrosis was observed on histopathological analysis compared with samples with less reticulation (mean score of 1.6 vs. 1.0, p=.040), accompanied by inflammation in all samples.

Severe ground-glass opacification (≥40% core volume, n=8/36) did not correlate with the grade of fibrosis (no fibrosis, n=2; mild para-septal fibrosis, n=6). Inflammation was present in all but one sample with severe ground-glass opacification (n=7/8, 87.5%) while inflammation was present in 24 of the 28 samples (86%) without severe ground-glass opacification. Vasculopathy was present independently of the presence or grade of fibrosis and was not associated with any of the radiological estimations outlined above.

## Discussion

In the present study, we investigated the radiological and histopathological correlates of ILAs. We demonstrated that ground-glass opacification, reticulation and bronchiectasis were the most frequent findings on ex-vivo CT in lung specimens with ILAs. Para-septal fibrosis (78% of samples) and accompanying (lymphocytic) inflammation (86% of samples) were most frequently observed via histopathological assessment. We have furthermore demonstrated CT underestimated the degree of fibrosis in relatively normal appearing lung regions with histopathologic evidence of moderate fibrosis (8 of 11 samples) and inflammation (9 of 11 samples). The presence of reticulation on CT was associated with more extensive fibrosis on histopathology.

In this study, the most prominent findings in early-stage ILD included (I) para-septal fibrosis, (II) lymphocytic inflammation, (III) bronchiectasis and (IV) some degree of vasculopathy, all of which will be discussed in more detail below.

### Para-septal fibrosis

this was most frequently observed in 28 of 30 samples exhibiting fibrosis, indicating that fibrosis seems to originate from the periphery of the secondary pulmonary lobule, moving inwards to the interstitial and centrilobular areas. This was also observed in our previous study investigating radiologically normal appearing areas in end-stage IPF lungs (9). We found that opacities in or near the interlobular septa were higher or enlarged, which causes reticulation, and ultimately honeycombing, as airway-like structures along the opacities enlarge and gradually fill the entire secondary pulmonary lobulus, giving rise to the typical honeycomb appearance which was not yet visible in this cohort of early ILD lungs.

### Lymphocytic inflammation

the role of inflammation in IPF genesis and progression remains controversial. Inflammation is believed to be induced by the ongoing fibrosis, mostly due to the observation that anti-inflammatory trials were largely negative (11). Therefore, it is of interest that predominantly lymphocytic inflammation was found in a large proportion of the samples in our study, and moreover, inflammation was visible before the onset of true fibrosis. This agrees with a recent study by Luzina et al investigating the transcriptome of morphologically inconspicuous areas in IPF lungs, which found a strong immune and inflammation-related signature, specifically of activated lymphocytes (12). However, this was only investigated in end-stage lungs where the entire micro-environment is likely confounded by the universal presence of fibrotic remodeling in various stages and degrees, but where, nevertheless, different gene expression signatures are found in inconspicuous rather than fibrotic areas of parenchyma (13,14). The predominant pattern of fibrosis and inflammation also seem to go hand in hand, indicating their intriguing relationship. Our finding of lymphocytic inflammation in early disease may be of primary importance in ILD.

### Bronchiectasis

We also observed radiologic evidence of bronchiectasis in five/eight specimens, while the corresponding histopathology demonstrated bronchiectasis in 14/36 samples and peri-bronchial fibrosis in 15/36 samples. Hida et al (15) demonstrated that 285 of 378 patients (75%) with ILAs showed some degree of bronchiectasis, which was associated with a worse outcome compared with patients with ILAs without bronchiectasis. Similarly, prognosis was worse in patients where bronchiectasis progressed over time (16). Consistently higher degrees of airway wall thickness have also been found in patients with ILAs and IPF (17), thereby supporting a pathophysiological role of the airways in disease progression. This is in line with our own data indicating that small airways are decreased by 57% in lung explants with IPF, even in areas which are only minimally affected by fibrosis (6). The importance of the airways in IPF is further corroborated by biological evidence that aberrant basal cells, the most important airway progenitor cell with the ability to proliferate and differentiate into virtually all epithelial cell types of the airway epithelium, have been found to be dysregulated in IPF (18). Our histopathological assessment of ILA lungs also showed important peri-bronchial inflammation (25% of samples) and peri-bronchial fibrosis (42% of samples) in early disease specimens, further suggesting that the airways might play an important role in the pathophysiology of IPF.

### Vasculopathy

The role of the (micro-) vasculature remains elusive in IPF/ILD. However, emerging data shows an important role for vascular changes in the process of pulmonary fibrosis. Recent single cell sequencing data identified a new type of vascular endothelial cell that might be involved in the progression of fibrosis in IPF (19). Along these lines, Ackermann et al provided compelling evidence of increased intussusceptive angiogenesis, a rapid process of blood vessel neoformation by splitting of a vessel into two lumens through incorporation of circulating angiogenic cells, in specific subtypes of ILD (20). Changes in the vessel-related structures, a computer-derived CT variable, is also a strong predictor of outcome in IPF (21).

By design and methodology, our study has limitations. Our specific comprehensive analytic approach precluded the analysis of many samples, and the availability of fresh whole lungs/lobes in such early stages of disease is limited. Prior studies included a matched control group revealing that substantial fibrosis can be observed in samples that show no radiological abnormalities; no control study was available in our study. Due to the anonymity of the organ donation procedure we do not have access to certain clinical variables such as pulmonary function, smoking status or the ethical permission to study genetic mutations (e.g. MUC5B or telomere changes).

In conclusion, we have demonstrated that early histologic features of lung fibrosis, primarily encompassing para-septal fibrosis, lymphocytic inflammation and bronchiectasis, are present even in normal-appearing lung regions on CT. Future studies are required to further refine and validate the concept of interstitial lung abnormalities and their relation to the genesis and progression of interstitial lung disease.

## Supplementary Material

Supplementary Material

## Figures and Tables

**Table 1:** Participants Characteristics and Radiologic Scoring

Age (y)	Sex	Smoking	Source of tissue	Extent of disease	Localization	Reticulation	GGO	BRECT	Emphysema
60	M	Unknown	Unused donor R	10-20%	Lower lobe	x	x	x	
60	M	Unknown	Unused donor L	5-10%	Lower lobe	x	x		
65	W	30 pack years	Tumor resection LLL	10-20%	Lower lobe	x	x		
69	M	Unknown	Unused donor L	10-20%	Lower lobe	x	x	x	x
70	M	Never smoker	Unused donor R	5-10%	Diffuse	x	x	x	x
76	M	20 pack years	Tumor resection RLL	10-20%	Lower lobe	x	x	x	
83	W	Never smoker	Unused donor R	20-30%	Diffuse	x	x		
83	W	Never smoker	Unused donor L	20-30%	Diffuse	x	x	x	

Note.— M=Male; W=Woman; R=right; L=left, LLL= left lower lobe; RLL= right lower lobe; GGO= ground-glass opacification; BRECT= bronchiectasis.

**Table 2:** Overview of the Main Histologic Findings of Lungs with Interstitial Lung Abnormalities

Histologic feature	n (%)
Fibrosis present	30 (83%)
Peri-bronchial	2 (6%)
Interstitial	0 (0%)
Para-septal	6 (17%)
Peri-bronchial and para-septal	3 (8%)
Para-septal and interstitial	9 (25%)
All compartments	10 (28%)
Lymphocytic inflammation	31 (86%)
Peri-bronchial	2 (6%)
Interstitial	7 (19%)
Para-septal	5 (14%)
Peri-bronchial and para-septal	2 (6%)
Para-septal and interstitial	10 (28%)
All compartments	5 (14%)
Vasculopathy	12 (33%)
Bronchiectasis	14 (39%)
Fully developed fibroblast foci	3 (8%)
Incomplete fibroblast foci	4 (11%)

Note. A total of 36 samples from 7 lungs were included for further histologic processing.

## Data Availability

Data generated or analyzed during the study are available from the corresponding author by request.

## Summary statement

The histopathological features of interstitial lung abnormalities included paraseptal organized fibrosis and lymphocytic inflammation that were present in regions considered normal on CT.

## Key Results

- In eight explant lungs/lobes with interstitial lung abnormalities (ILAs) from 6 donors, histopathologic analysis showed varying degrees of fibrosis in areas that appear normal on CT.
- Fibrosis in lungs with ILAs was organized para-septally and was associated with predominant lymphocytic inflammation.
- Vasculopathy and bronchiectasis were also often present on histopathological examination of lungs with ILAs, while fully developed fibroblastic foci were only rarely observed.
