# Supplementary material for: Radiologic and histologic correlates of early interstitial lung changes in explant lungs
Source: Radiology. Author manuscript; Available in PMC 2023 Apr 1. (PMC7614383; doi:10.1148/radiol.221145)
Supplement: Supplementary Material [file EMS156834-supplement-Supplementary_Material.docx]

|  |  |  | Radiological assessment | | | Histopathologic assessment | | | | | |
| --- | --- | --- | --- | --- | --- | --- | --- | --- | --- | --- | --- |
|  | ID | Core n° | Normal | Reticulation | GGO | BRECT | fibrosis severity | location fibrosis | location lymphocytic inflammation | vasculopathy | fibroblast foci |
|  | Lung 1 | core 1 | 95 | 0 | 5 | no | 0 | n a | n a | no | no |
|  | Participant 1 | core 2 | 60 | 25 | 15 | no | 1 | paraseptal / interstitial | n a | no | no |
|  |  | core 3 | 75 | 20 | 5 | no | 1 | peribronchial | n a | no | no |
|  |  | core 4 | 75 | 15 | 10 | no | 2 | paraseptal / interstitial | paraseptal / interstitial | no | no |
|  |  | core 5 | 60 | 30 | 10 | yes | 1 | peribronchial | peribronchial | no | no |
|  |  |  |  |  |  |  |  |  |  |  |  |
|  | Lung 2 | core 1 | 25 | 55 | 20 | no | 1 | paraseptal / interstitial | paraseptal / interstitial | no | no |
|  | Participant 2 | core 2 | 80 | 15 | 5 | no | 2 | peribronchial / paraseptal / interstitial | peribronchial / paraseptal / interstitial | no | no |
|  |  | core 3 | 20 | 25 | 55 | no | 2 | paraseptal | peribronchial | no | no |
|  |  | core 4 | 35 | 25 | 40 | no | 1 | paraseptal / interstitial | paraseptal / interstitial | no | yes |
|  |  | core 5 | 75 | 10 | 15 | no | 0 | n a | interstitial | no | no |
|  |  |  |  |  |  |  |  |  |  |  |  |
|  | Lung 3 | core 1 | 20 | 40 | 40 | no | 1 | peribronchial / paraseptal | interstitial | no | no |
|  | Participant 3 | core 2 | 15 | 20 | 65 | no | 2 | peribronchial / paraseptal / interstitial | peribronchial / paraseptal / interstitial | yes | yes (incomplete) |
|  |  | core 3 | 15 | 20 | 65 | no | 0 | n a | n a | yes | no |
|  |  | core 4 | 75 | 25 | 0 | no | 0 | n a | interstitial | tes | no |
|  |  | core 5 | 30 | 50 | 20 | yes | 2 | peribronchial / paraseptal / interstitial | interstitial | no | no |
|  |  | core 6 | 30 | 15 | 55 | no | 2 | peribronchial / paraseptal / interstitial | interstitial | yes | no |
|  |  |  |  |  |  |  |  |  |  |  |  |
|  | Lung 4 | core 1 | 30 | 40 | 30 | no | 0 | n a | paraseptal / interstitial | no | no |
|  | Participant 4 | core 2 | 30 | 40 | 30 | no | 1 | paraseptal | interstitial | no | no |
|  |  | core 3 | 60 | 35 | 5 | no | 1 | paraseptal | paraseptal | no | no |
|  |  | core 4 | 65 | 15 | 20 | yes | 1 | paraseptal | paraseptal | no | no |
|  |  | core 5 | 70 | 20 | 10 | no | 1 | paraseptal / interstitial | interstitial | no | no |
|  |  |  |  |  |  |  |  |  |  |  |  |
|  | Lung 5 | core 1 | 40 | 20 | 40 | yes | 1 | paraseptal / interstitial | paraseptal / interstitial | no | no |
|  | Participant 5 | core 2 | 0 | 80 | 20 | yes | 2 | peribronchial / paraseptal / interstitial | peribronchial / paraseptal / interstitial | yes | no |
|  |  | core 3 | 10 | 75 | 15 | yes | 2 | peribronchial / paraseptal / interstitial | peribronchial / paraseptal / interstitial | no | no |
|  |  | core 4 | 70 | 20 | 10 | yes | 1 | paraseptal (focal) | paraseptal | no | no |
|  |  | core 5 | 60 | 25 | 15 | no | 1 | paraseptal | paraseptal | no | no |
|  |  |  |  |  |  |  |  |  |  |  |  |
|  |  |  |  |  |  |  |  |  |  |  |  |
|  | Lung 6 | core 1 | 10 | 75 | 15 | no | 1 | peribronchial / paraseptal / interstitial | paraseptal / interstitial | yes | yes |
|  | Participant 5 | core 2 | 20 | 50 | 30 | yes | 1 | peribronchial / paraseptal | peribronchial / paraseptal | no | no |
|  |  | core 3 | 10 | 65 | 25 | yes | 2 | peribronchial / paraseptal / interstitial |  | no | no |
|  |  | core 4 | 25 | 35 | 40 | yes | 0 | n a | paraseptal | yes | no |
|  |  | core 5 | 85 | 0 | 15 | yes | 1 | peribronchial / paraseptal | paraseptal | no | no |
|  |  |  |  |  |  |  |  |  |  |  |  |
|  | Lung 7 | core 1 | 35 | 35 | 30 | no | 2 | peribronchial / paraseptal / interstitial | paraseptal / interstitial | yes | yes (incomplete) |
|  | Participant 6 | core 2 | 85 | 0 | 15 | yes | 1 | peribronchial / paraseptal / interstitial | paraseptal / interstitial | yes | yes |
|  |  | core 3 | 85 | 10 | 5 | no | 1 | paraseptal / interstitial | paraseptal / interstitial | yes | yes (incomplete) |
|  |  | core 4 | 35 | 40 | 25 | yes | 1 | paraseptal / interstitial | n a | yes | no |
|  |  | core 5 | 65 | 10 | 25 | yes | 1 | paraseptal / interstitial | peribronchial / paraseptal | yes | yes (incomplete) |

Supplemental table 1: core-specific pathologic and radiologic information
